# Cellular Crosstalk in the Vascular Wall Microenvironment: The Role of Exosomes in Vascular Calcification

**DOI:** 10.3389/fcvm.2022.912358

**Published:** 2022-05-23

**Authors:** Yun-Yun Wu, Su-Kang Shan, Xiao Lin, Feng Xu, Jia-Yu Zhong, Feng Wu, Jia-Yue Duan, Bei Guo, Fu-Xing-Zi Li, Yi Wang, Ming-Hui Zheng, Qiu-Shuang Xu, Li-Min Lei, Wen-Lu Ou-Yang, Ke-Xin Tang, Chang-Chun Li, Muhammad Hasnain Ehsan Ullah, Ling-Qing Yuan

**Affiliations:** ^1^Department of Metabolism and Endocrinology, National Clinical Research Center for Metabolic Diseases, The Second Xiangya Hospital, Central South University, Changsha, China; ^2^Department of Radiology, The Second Xiangya Hospital, Central South University, Changsha, China; ^3^Department of Nuclear Medicine, Xiangya Hospital of Central South University, Changsha, China; ^4^Department of Pathology, The Second Xiangya Hospital, Central South University, Changsha, China

**Keywords:** exosomes, vascular calcification, vascular smooth muscle cells, endothelial cells, immune cells

## Abstract

Vascular calcification is prevalent in aging, diabetes, chronic kidney disease, cardiovascular disease, and certain genetic disorders. However, the pathogenesis of vascular calcification is not well-understood. It has been progressively recognized that vascular calcification depends on the bidirectional interactions between vascular cells and their microenvironment. Exosomes are an essential bridge to mediate crosstalk between cells and organisms, and thus they have attracted increased research attention in recent years. Accumulating evidence has indicated that exosomes play an important role in cardiovascular disease, especially in vascular calcification. In this review, we introduce vascular biology and focus on the crosstalk between the different vessel layers and how their interplay controls the process of vascular calcification.

## An Introduction to Exosomes

Exosomes, with a diameter of 30–150 nm, are a type of extracellular vesicle (EV) released from intracellular stones called multivesicular bodies (MVBs). They were first reported by Johnstone (1) and simply considered a pathway to expel waste to regulate membrane recycling during cell proliferation. Exosomes have been shown to play a role in communicating B lymphocytes and dendritic cells (2). Secreted exosomes were able to regulate cellular functions locally or even over long distances. Besides, exosomes could act as messengers that transfer lipids, messenger RNAs (mRNAs), microRNAs (miRNAs), and proteins between cells (3). It has become clear that the conditioned culture media as well as body fluids including blood, urine, and cerebrospinal fluid contain numerous cell-derived vesicles. Exosomes played prominent roles in tumor metastasis, bone diseases, diabetes, obesity, and thyroid disease, among other conditions. Thus, there is growing interest in understanding the functional role and regulatory mechanisms of exosomes (4–8).

Exosomes are derived from plasma membrane invagination into an early endosome. Then, the mature endosome can evolve into MVBs with different cargo, followed by the budding of selective intracellular vesicles from intraluminal vesicles (ILVs). However, there is no consensus regarding whether these vesicles are involved in general or specific physiological secretion because different types of membrane vesicles could be generated in different conditions (9, 10). Ectosomes/microparticles (MPs), and apoptotic bodies (ABs) are other common EVs, which have often been confused with exosomes (11, 12). However, their diameter, assembly, subcellular origin, sedimentation force, and biogenetic mechanisms are different than exosomes (11). We described the characteristics of different types of EVs in a previous publication (5). In addition, some researchers have proposed using the term “extracellular vesicles” rather than “exosomes,” because the components, classification, and biological functions of these vesicles are unclear (13).

Cardiovascular disease remains the leading cause of morbidity and mortality throughout the world. In recent years, exosomes haves gained increasing attention in the cardiovascular field, largely owing to their ability to transmit biological information between adjacent and distant cells (14). Previous reviews mostly focused on the underlying mechanisms of exosomes in cardiovascular diseases (15, 16), yet ignore the cellular crosstalk among different vessel layers. Recent studies have found that endothelial cells, VSMCs, fibroblast, macrophages, and even circulation blood cells around vascular wall environments respond actively to stimulus and participate in vascular calcification *via* transdifferentiating, cytokine secretion, extracellular vesicle synthesis, angiogenesis regulation, and hemodynamics. In this article, we aim to summarize the complex interactions between exosomes derived from multiple cell populations in the cardiovascular microenvironment.

## Definition and Classification of Vascular Calcification

Vascular calcification (VC) is a life-threatening complication characterized by ectopic calcium-phosphate deposition in the blood vessel walls. VC has been reported as an independent predictor of all-cause mortality and cardiovascular mortality in patients with uraemia, and it is inextricably associated with cardiovascular events including myocardial infarction, cerebral embolism, and amputation (17, 18). The clinical classification of VC depends on the area of calcification, mainly involving intimal calcification, medial calcification, adventitial calcification, and valve calcification (17, 19, 20). Intimal calcification is common in patients with atherosclerosis, also known as atherosclerotic calcification. Under the forces of inflammation, oxidative stress, mechanical stress, and other factors, the intima often proliferates with lipid deposits and macrophage infiltration, which present as focal spots or plaques (21). Vascular medial calcification, also known as Mönckeberg's sclerosis, occurs in elderly individuals as a result of aging, while it is often accelerated in patients with metabolic disorders, such as diabetes, chronic kidney disease (CKD), and heritable mineralization disorders (22). Medial calcification can occur independently of atherosclerotic lesions, resulting in increased arterial stiffness, reduced compliance of the blood vessels, and elevated risk of adverse cardiovascular events and mortality (23). In turn, calcification in different locations have their own distinctive features, with intimal and medial calcification being most pronounced.

As we illustrate above, VC is able to occur in different layers of the vessel wall and highly correlated with major adverse cardiovascular events, so several treatments aim to target anti-VC. However, effective therapy is still lacking. A meta-analysis showed that statin has a strong association with coronary stenoses rather than coronary calcification (24). Besides, Pi binders didn't work in reducing VC in the CKD population (25). Because there is no specific treatment available for VC currently, a better understanding of the cellular crosstalk that occurs during VC could provide new biomarkers for diagnosis and prognosis as well as novel targets for treatment.

## Mechanisms of Vascular Calcification

Early studies had suggested that VC is a passive pathologic process in the vascular system due to imbalanced calcium and phosphorus metabolism (26). However, since the late 1990s researchers have gradually recognized that VC is an active, multifaceted, and dynamic process that is similar to bone development (27). The phenotype transition of vascular smooth muscle cells (VSMCs) into osteoblastic cells is the key step that contributes to VC progression. Oxidative stress, inflammation, apoptosis, autophagy, matrix remodeling, and microRNAs (28, 29) have also been reported to play important roles in regulating VC. Exosomes are of great significance in regulating cardiovascular homeostasis and bone biology (30). Under physical states, vascular cells secret exosomes to modulate the normal function of the vascular wall. However, certain exosomes gain calcification potential after the microenvironment is disrupted, and thus contribute to VC procession through transport and loading (31). Latest studies have shown that EVs derived from lipopolysaccharide (LPS-EK)-treated macrophages can propagate inflammation and oxidative stress in VSMCs, thus facilitating the progression of VC (32). Moreover, it is reported that bone marrow mesenchymal stem cell-derived exosome (BMSC-Exo) acts as an anti-calcification effector in CKD by delivering enclosed miR-381-3p. In summary, exosome plays an irreplaceable role in vascular calcification.

## Cell Biology in the Vascular Wall Microenvironment

**Figure 1:**
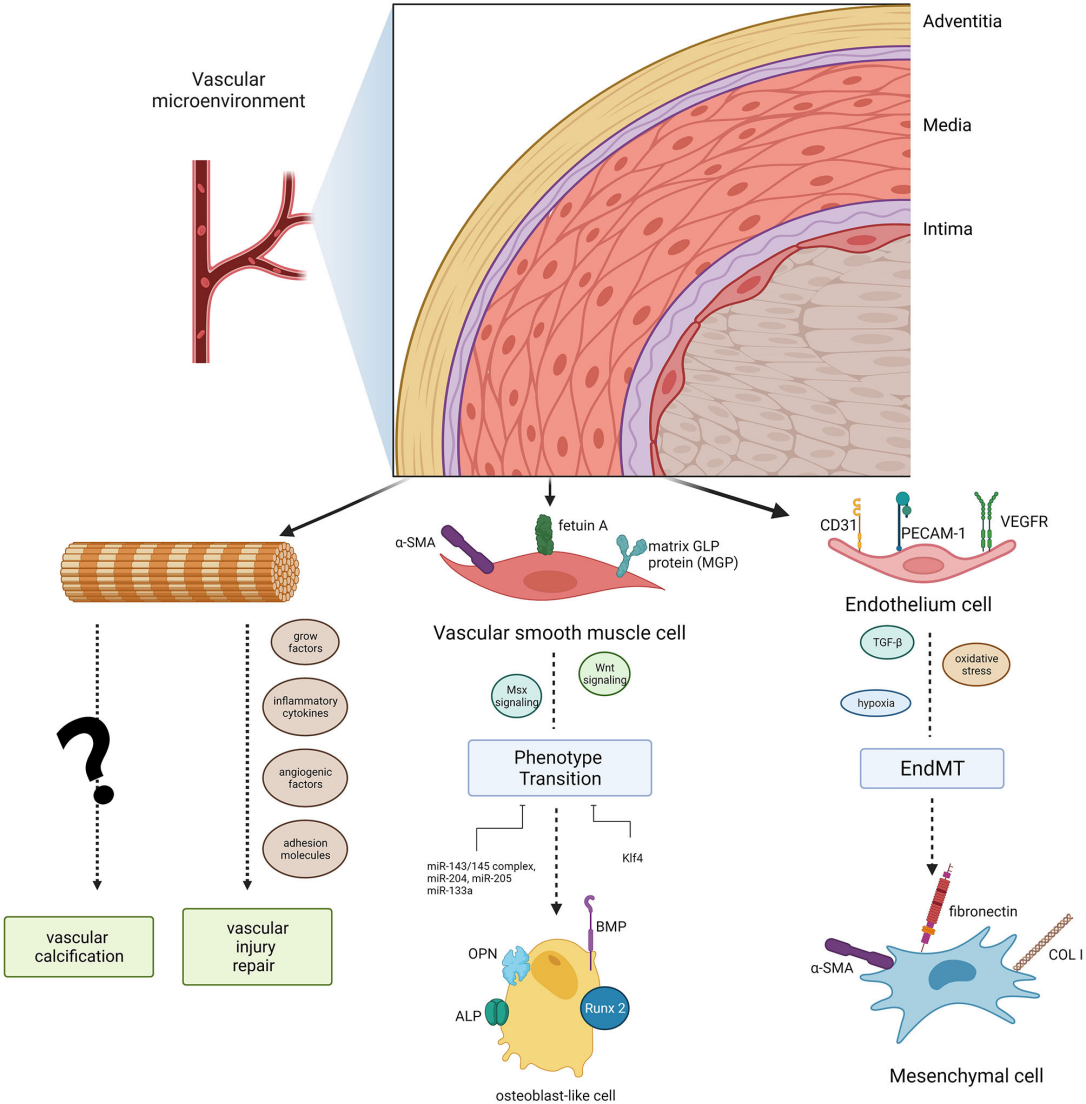
Diagram of the cell populations in the vascular microenvironment and their roles in vascular calcification. Endothelial cells (ECs) and vascular smooth muscle cells (VSMCs) can induce phenotype transitions (EndMT, endothelial- mesenchymal transitions, a specific transition of ECs) in the microvasculature mineralization process *via* several signaling pathways. Adventitial fibroblasts could function in vascular calcification as well as vascular injury repair.

Anatomically, blood vessel walls can be divided into three layers: tunica intima, tunica media, and tunica adventitia. However, the vessel wall is not so divisive as it seems, it is more inclined to function as an integrated whole with cellular crosstalk, thus we regard it as “vascular wall microenvironment.” The vascular wall microenvironment comprises various cell types, including cells embedded into vessel walls, blood cells, stromal cells, and immune cells. These cells function together to convey blood throughout the body, bringing nutrients, removing wastes (33), and modulating vascular tone and blood pressure. Hence, maintaining normal metabolic crosstalk among multiple cell types in different layers is critical for vascular wall microenvironment stabilization and remodeling. In this section, we comprehensively summarize the cell biology in different vessel layers and the evidence that they act as key mediators and regulators of VC in the vascular wall microenvironment (Figure 1).

### Tunica Intima: Endothelial Cells

Endothelial cells (ECs) are the principal cells of the thinnest intimal layer. The long axis of ECs is consistent with the direction of blood flow, providing a smooth surface for blood flow and building a barrier between the central lumen and the surrounding vessel wall. Hence, it is not surprising that ECs have the ability to initiate both haemostasis and inflammation (34) and to modulate blood pressure and angiogenesis. More importantly, ECs are the first cells of the vascular system exposed to harmful stimuli. ECs can transfer messages *via* secretion of pro-calcification cytokines, such as vascular endothelial growth factor (VEGF), platelet-derived growth factor (PDGF), basic fibroblast growth factor (bFGF), insulin-like growth factor 1 (IGF-1), nitric oxide (NO), prostacyclin (PGI2), and endothelin (ET), among others. Furthermore, ECs initiate the endothelial–mesenchymal transition (EndMT) in the microvasculature mineralization process by altering its EC-specific markers into mesenchymal cell-specific markers (35). In fact, ECs are far more than just a physical barrier: they are extensively involved in VC (36). The exosome-mediated crosstalk between ECs and VSMCs, through a paracrine mechanism, likely plays a regulatory role in VC pathogenesis.

### Tunica Media: VSMCs

The tunica media is an elastic membrane mostly composed of VSMCs. These cells are required for optimal functioning of arteries, primarily facilitating vessel dilation, and constriction to direct blood flow into various organs and tissues (37–39). VSMCs are arranged in a spiral form around the vessel lumen and display great phenotypic plasticity (40). Under pathological conditions, such as vascular injury, mechanical stretch, and other stimulation (41–43), a physical contractile phenotype could differentiate toward a synthetic phenotype to remodel extracellular matrix components. VSMCs lose the contractile phenotype-specific markers α-smooth muscle actin and smooth muscle 22 alpha (SM22 alpha) and progressively remodel themselves into osteoblast-like cells that express specific osteogenic genes in VC progression, such as Runt-related transcription factor 2 (RUNX2), osteopontin (OPN), osteocalcin (OCN), bone morphogenetic protein-2 (BMP-2), alkaline phosphatase (ALP), and osterix (44, 45). Activation of downstream Msx2 and Wnt signaling initiates VSMC calcification (46, 47). Recent studies have even indicated that there was great heterogeneity in VSMC phenotypes, which possessed a variety of features, including adipocytic, senescent, foam, and osteochondrogenic phenotypes (48–50). Epigenetic regulation (51) and microRNAs also contribute to VSMC differentiation under calcifying conditions (30, 52). These novel findings that link VSMC and tunica media VC have important implications for the design of novel diagnostic and therapeutic strategies. However, these studies have only focussed on the direct effects of factors on VSMCs and have largely ignored the combined effects of the vascular wall microenvironment. An interesting perspective on triggered exosome excretion within the vascular wall microenvironment has received more attention (22, 46).

### Tunica Adventitia

The tunica adventitia mainly consist of fibroblasts, myofibroblasts, immune cells (macrophages, dendritic cells, T cells, B cells, and mast cells), stem/progenitor cells, vascular pericytes, smooth muscle cells, adipocytes, among others; hence, the adventitia is the most complex layer of blood vessels (53). The cells in the adventitia can regulate the structure and function of all three layers of the vascular wall (54).

There is an intriguing hypothesis that adventitial cells are “sentinel cells” of vascular dysfunction, carrying out a passive secondary response to monitoring the vascular wall microenvironment (55). Adventitial fibroblasts and their subpopulations, which are recognized as adipogenic progenitors, could transdifferentiate into myofibroblasts (56). Researchers have shown that in diabetic mice feed with a high-fat diet, myofibroblasts in the aortic adventitia participate in osteogenesis, targeting vascular Msx1 and Msx2 (57). Besides, adventitial fibroblasts respond quickly to the release of growth factors, inflammatory cytokines, angiogenic factors, adhesion molecules, and reactive oxygen species when faced with vascular injury and stress (53). Moreover, the adventitial progenitor cells contribute to both vascular injury repair and VC (53), confirming their indispensable role in VC. However, it is unclear whether adventitial stem cells only proliferate and differentiate to maintain vascular wall microenvironment homeostasis, or whether they also have the ability to reprogram vascular development under pathological conditions.

## Exosome-Mediated Crosstalk in the Vascular Wall Microenvironment

Numerous studies have demonstrated that exosomes mediate changes in the vascular wall microenvironment, including facilitating hydroxyapatite crystal deposition (56), regulating phenotype transition (58), or interfering in underlying signaling. In the vascular system, exosomes can be released by several types of cells, and their biological effects on the recipient cells depend on exosomes and the microenvironment where the recipient cells reside. In this section, we summarize exosome-mediated crosstalk among cells during VC in the following paragraphs.

### VSMCs–VSMCs Crosstalk

VSMCs are major effector cells of VC. It has long been recognized that VSMCs can transfer pro-calcification signals among themselves by releasing matrix vesicles (MVs). MVs in blood vessels resemble bone MVs, which are the initial sites for mineralization (59–62). Bone MVs are rich in calcium phospholipid-binding proteins, which transform excess extracellular calcium into calcified MVs. Calcified MVs trigger hydroxyapatite crystal deposition in the extracellular matrix and then interact with collagen, which promotes bone mineralization (63–65).

Similarly, MVs played an important role in the initiation of vascular mineralization by regulating calcium and phosphate homeostasis in the extracellular matrix (66, 67). Reynolds et al. (68) demonstrated that MVs derived from VSMCs contain mineralization inhibitors such as fetuin-A, an endogenous calcium binder, to prevent VC under normal conditions (58). This was consistent with our previous research that plasma vesicles derived from patients with uremia and renal transplant recipients with VC had different contents of endogenous calcification inhibitors or promoters (69). High circulating endogenous calcium and phosphate might contribute to enhancing VC by decreasing the mineralization inhibitor matrix Gla protein (MGP) (68, 70). Utilization of the L-type calcium channel blocker verapamil could inhibit vascular smooth muscle mineralization and MVs activity (71). This evidence suggested that MVs mainly take function *via* regulating calcium and phosphate metabolism.

Given that MVs share similar compositional characteristics to exosomes, new discoveries are shedding light on the working mechanisms of exosomes (66). Exosomes can act as a specific signal transporter participating in cell proliferation, migration, apoptosis, calcification, and differentiation through VSMC–VSMC crosstalk. Notably, VSMC calcification was mediated by regulating the volume of exosomes secreted from donor VSMCs in response to environmental factors. For example, environmental calcium stress could increase exosome release from VSMCs, which subsequently promotes VC (66). Bhat et al. (72) also indicated that *Mcoln1* gene deletion in mice increases exosome secretion from VSMCs, which contributes to arterial tunica media calcification and stiffening. Other evidence showed that excessive reactive oxygen species (ROS) production, increased the number of exosomes released from VSMCs, therefore increasing the calcification of recipient VSMCs (67). Taken together, calcification of arteries was extended from calcified VSMCs to adjacent normal cells by regulating exosome excretion, and the detailed mechanisms should be elucidated further.

Exosomes are able to carry important cargo such as mRNAs, miRNAs, lipids, and proteins from donor cells and thus regulate the function of recipient cells (30). Hence, besides modifying the number of released exosomes, the progression of calcification could be controlled by altering the content of functional molecules in exosomes. Pan et al. (73) illustrated that microRNA profiles were significantly altered in exosomes from VSMCs undergoing calcification induction. Nine hundred eighty-seven and 92 significantly upregulated and downregulated miRNAs were identified, respectively. Other research had revealed that miRNA expression in MVs during VC altered the expression of osteogenic markers (Smad1, Runx2, ALP, and osterix) (74–77), providing strong support that exosomes could transport vital information during VC. In a recent study, we showed that melatonin treatment diminished both calcification and senescence of VSMCs. The anti-calcification role was mediated by decreased miR-204/miR-211 in exosomes released from calcified VSMCs (78). Clinically, circulating as well as cell-derived exosomes with a low fetuin-A level play a detrimental role in VC (58, 79). While the GFOGER peptide, a six amino-acid repeat present in type 1 collagen, alleviated VC by reducing the content of osteogenic switching–related proteins, such as TANK-binding kinase 1 and casein kinase II, in VSMC-derived EVs (80). These pieces of evidence remind us that the cargo loaded by exosomes secreted from VSMCs changes dynamically with the progression of VC, which provides potential diagnostic biomarkers and targets for treatment. However, more in-depth exploration needed to be conducted before applying in clinical.

A number of studies have focussed on the roles of exosomes in mineral deposition during VC. This focus has limited discoveries of how exosomes function as information transporters among cell populations (66). Nevertheless, there was a view that exosomes were just erroneously trapped in the extracellular matrix, which adds a new dimension to EV-mediated calcium deposition (81). It seemed that exosomes have more effects than what has been reported; thus, greater attention is needed regarding their role in VC. Clarifying how exosomes mediate cell-to-cell communication and the composition or phenotype of exosomes in physiological and pathological vascular conditions will be crucial to expanding our understanding of how exosome-mediated actions in vessels contribute to VC. This information could facilitate the discovery of new therapeutic targets and preventive strategies.

### ECs–VSMCs Crosstalk

Exosome-mediated crosstalk between ECs and VSMCs is widely recognized to be involved in regulating the formation of calcific plaque. Similar to VSMC–VSMC crosstalk, EC-derived EVs can mediate intercellular communication through distinct cargo and bioactive ligands. Exosomal miRNAs can convey information from the donor to the recipient to promote the phenotype transition of VSMCs in cardiovascular disease. Zernecke et al. (82) indicated that EC-derived membranous microvesicles convey paracrine alarm signals to recipient vascular cells to trigger the miR-126-mediated production of CXCL12 during atherosclerosis. Moreover, shear stress–stimulated EC-derived EVs containing miR-143/miR-145 controlled target gene expression in co-cultured VSMCs and led to atherosclerotic lesion diminishment in the aorta of *Apoe*^−/−^ mice (83). In elderly patients with CKD, endothelial microparticles (EMPs) from tumor necrosis factor-alpha (TNF-α)–stimulated human umbilical vein endothelial cells (HUVECs) loaded with BMP-2 could be actively taken up by VSMCs, thus inducing osteogenesis and calcification (84). Besides, Cavallari et al. (85) found that among the majority of circulating EVs derived from ECs, platelets, and monocytes/macrophages, only endothelial EVs showed notable enhancement in patients with CKD compared with healthy people, indicating that ECs are the major origin of circulating EVs in patients with CKD (85). Interestingly, compared with young subjects, only EVs from senescent ECs and the plasma of elderly people could induce calcification. These findings provide a foundation for elucidating the relationship between aging and EC-derived EVs (86). Furthermore, a recent study used fluorescence microscopy and confirmed the intracellular uptake of EVs between valvular interstitial cells to valvular ECs through the endosomal pathway, once again revealing the key role of EVs in cardiovascular cell-to-cell communication (81).

A series of studies have focussed on how exosome activity connects diabetes and VC. In one study, high glucose-induced diabetes-related VC/aging by increasing lactate dehydrogenase activity and the product of lipid peroxidation (denoted by the malondialdehyde content) through HUVEC-Exos (87). In another study, high glucose-stimulated ECs showed activation of the exosomal Notch3/mammalian target of rapamycin (mTOR) signaling pathway, and thus promoted VSMCs calcification/aging (88). These findings could lead to novel strategies for the prevention of diabetes induced VC. In summary, the investigation of exosomes from ECs revealed the relationship between endothelial cells and vascular calcification, thus providing innovative ideas for the intervention and treatment of vascular calcification. Of course, more in-depth exploration was required to be conducted.

### Immune Cells–VSMCs Crosstalk

Immune cells represent multiple cell populations that mediate a large part of the progression of various cardiac immune responses, such as atherosclerosis (89) and myocardial infarction. The immune response consists of innate and adaptive immunity, which has been considered a major contributor to the occurrence of cardiovascular disease in the past few years (89). Regulatory T (Treg) cells, a kind of cells that can negatively regulate the immune response, play a critical role in immune response. Exosomes are proven to contribute significantly to the homeostasis modulation of Treg cells by transmitting exosomal contents to recipient cells in diseased/healthy tissue (90). Previous studies have shown that Treg-cell-derived exosomes are suppressive effectors in the immune response by delivering miR-155 (91, 92). The key point is that miR-155 is of great significance in VC procession (77), and exosomal miR-155 derived from Treg cells might serve as another key source of miRs during VC. Therefore, exosomes are emerging as new biomarkers between immune response and VC procession, and such cellular crosstalk mostly depends on the miRs transportation.

Exosomes derived from immune cells and their parent cells have been shown to enhance pleiotropic dimensions of intercellular communication in some specific pathological states (93). New et al. (94) found that macrophage-derived MVs with exosomal markers (CD9 and TSG101) contributed directly to the early calcification of atherosclerotic plaques through the phosphatidylserine–annexin V-S100A9 membrane complex; this pathway could also be activated in diabetes mellitus (95). A novel mechanism is that high mobility group box 1 (HMGB1), a cytokine associated with biomineralization, promoted the secretion of MVs from macrophages and induced ectopic mineralization *in vitro* in a medium with high calcium and phosphorus *via* the RAGE/p38 MAPK/nSMase2 signaling pathway (96). An *in vivo* study indicated exosomes from cultured bone marrow dendritic cells (BMDCs) could be absorbed by aortic ECs of mice through intravenous injection (97). Furthermore, mature dendritic cell-derived exosomes activated endothelial inflammation and induced atherosclerosis *via* TNF-α-mediated activation of the NF-κB signaling pathway in a manner similar to that of lipopolysaccharide (97).

In addition to the direct cell-to-cell approach, immune cells can interfere in exosome-mediated information transport among other cells. In a macrophage/VSMC co-culture system, macrophage galectin-3 regulated the migration of VSMC-derived exosomes and induced diabetic vascular intimal/medial calcification translocation, which may provide a potential method for early intervention in diabetic VC (98). These findings have extended our knowledge of how exosomes derived from immune cells regulate immune responses during VC. This information has provided insights into searching for novel approaches to prevent VC.

### Stem Cells–VSMCs Crosstalk

Bone marrow-derived stem/progenitor cells are pluripotent, meaning they can differentiate into multiple stromal cell types, such as astrocytes, adipocytes, myocytes, fibroblasts, and osteoprogenitors (99, 100). Mesenchymal stromal cells (MSCs) play an essential role in vascular regeneration and development under physiological conditions because they can promote angiogenesis, mediate immunity, and restrain inflammation (101). Previous studies have mainly focussed on how cytokines released from MSCs modulate VC, and there have been only a few studies concerned with MSC-derived exosomes. Several compelling trials have focussed on how exosomes from bone marrow-derived MSCs are involved in VC. Exo treatment diminished the calcium content and alkaline phosphatase activity in the presence of high phosphorus due to exosomal microRNAs (102), such as miR-146a (103) and miRNA-126/miRNA-145 (104). Similar to other stem cells, hematopoietic stem cells (HSCs) are capable of secreting EVs, including exosomes and microvesicles. Based on the available data, the cardioprotective action of injected HSCs might be explained by paracrine communication of HSC-derived exosomes and microvesicles between donor and recipient cells. Moreover, the indirect cardioprotective functions of HSCs in cardiovascular disease are mainly due to the EVs produced in HSC-derived cells, such as dendritic cells and endothelial stem cells (105). Therefore, in-depth research of the molecular mechanism of the exosomes derived from stem cells in the vascular wall microenvironment is critical to take full advantage of the promising prospects of effective treatment.

### Other Cell Populations

Circulating microparticles (cMPs) also play essential roles in cellular crosstalk and act as messengers to activate cells. Suades et al. (106) reported that platelet-derived microparticles (pMPs) are able to stimulate thrombosis. They also found that high levels of TSP1+/CD142+ platelet-derived microparticles distinguish young patients with high cardiovascular risk and subclinical atherosclerosis from matched healthy volunteers (*P* < 0.0001) (106). Women with a history of pre-eclampsia show a high risk for future coronary artery calcification (CAC) and metabolic disorder, which may be associated with a larger group of specific blood-borne, cell-derived antigen-positive microvesicles, including stem/progenitor cell antigen CD117 and tissue factor, ICAM-1abundent microvesicles (107). Another interesting trial showed that EVs isolated from aortic adventitial fibroblasts in Wistar–Kyoto rats can deliver miR-155-5p to attenuate VSMC proliferation and vascular remodeling by suppressing angiotensin-converting enzyme (ACE) expression (108). These findings indicate the tremendous diagnostic and therapeutic potential of EVs-based cardiac regenerative therapies. However, their diagnostic potential for CAC remains to be determined.

**Figure 2:**
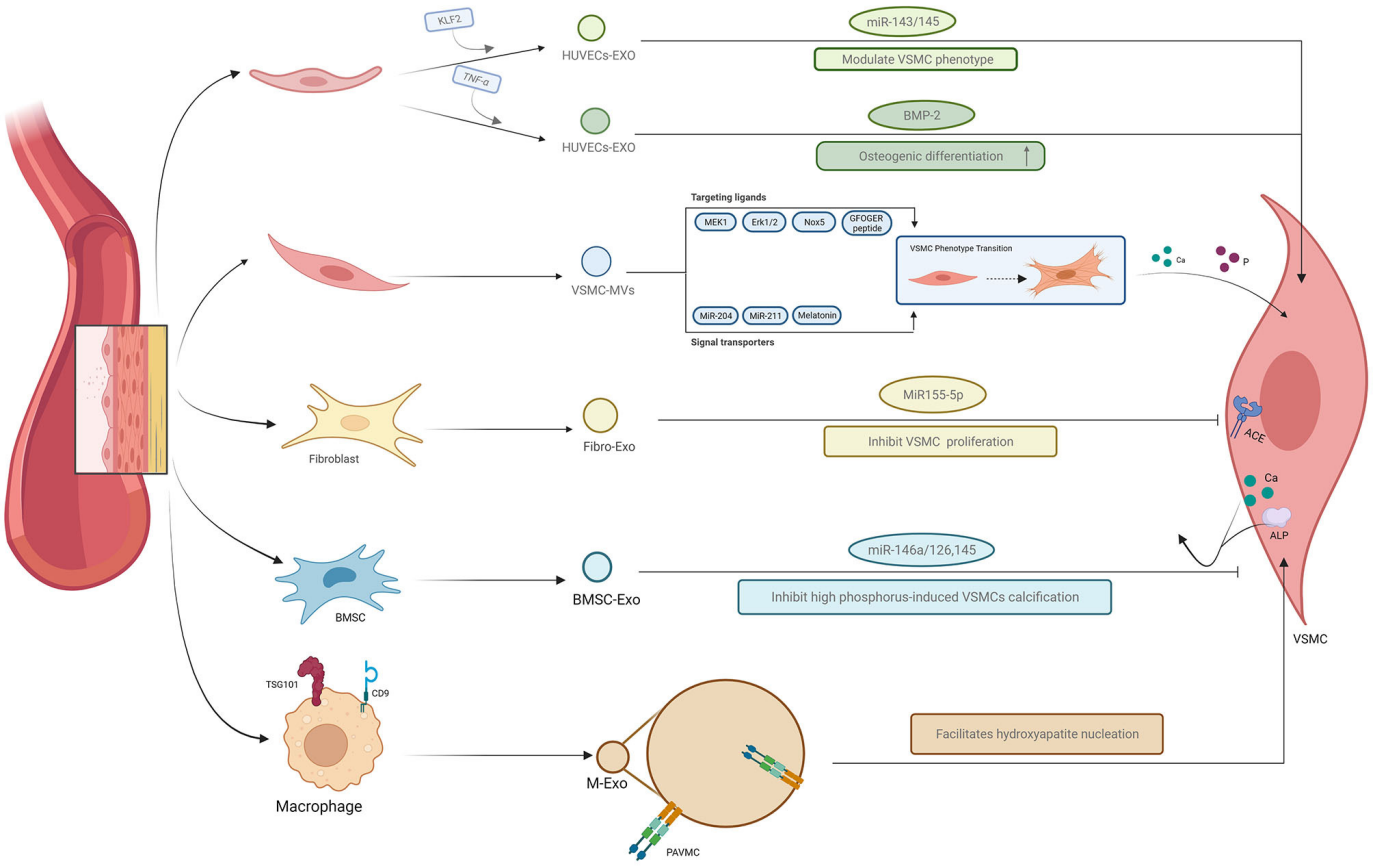
Exosome-mediated crosstalk in the vascular microenvironment. The exosomes derived from different cell populations can facilitate or inhibit vascular calcification *via* exosome-mediated crosstalk between VSMCs and other cell populations through different molecules, such as miRNAs and bone morphogenetic protein-2 (BMP-2), among others.

Collectively, it is easy to understand that the exosomes mediate the communication between different cells within the vascular wall microenvironment. A brief explanation of the relationship was summarized in Figure 2. And the summary of bioactive cargo in different exosomes and their regulatory pathway is presented in Table 1.

**Table 1:** Role of exosomes in mediating vascular calcification in vascular wall microenvironment.

**Exosome origin**	**Cargo**	**Involved pathway**	**Effects on vascular calcification**	**References**
Vascular smooth muscle cells (VSMCs)	Mineralization inhibitors (fetuin-A)	Mitogen-activated protein kinase (MEK1 and ERK1/2) signaling	Decrease intracellular Ca^2+^ of recipient VSMCs	(79)
	Ca^2+^	NADPH oxidase 5 (Nox5) mediated reactive oxygen species (ROS) production	Increase cytosolic Ca^2+^ and VSMC phenotypic switching	(67)
	Proteins involved in vascular calcification (such as osteogenic markers, TANK-binding kinase 1, and casein kinase II)	GFOGER peptide (a specific, six amino-acid repeat in type 1 collagen)	Decrease osteogenic switching in VSMCs	(80)
	miR-204/miR-211 cluster	Melatonin-mediated paracrine action	Attenuate the osteogenic differentiation and senescence of VSMCs	(78)
Endothelial cells	Apoptotic bodies	miR-126-mediated CXCL12 production	Confer features of plaque stability in different mouse models of atherosclerosis	(82)
	miR-143/miR-145	Krüppel-like factor 2 (KLF2)-transduced pathways	Reduce atherosclerotic lesion formation	(83)
	Bone morphogenetic protein 2 (BMP-2)	Upregulation of Cbfa1 and downregulation of SM22α	Enhance VSMC osteogenesis and calcification	(84)
Macrophages	S100A9 and annexin V	Facilitate hydroxyapatite nucleation	Accelerate microcalcification in chronic renal disease	(95)
	HMGB1	RAGE/p38 MAPK/nSMase2 signaling pathway	Lead to mineral deposition	(96)
	Pro-inflammatory cytokines and CAD, PAI-1, and Saa3 proteins	Inflammatory and oxidative responses.	Aggravate the VC process	(32)
Mesenchymal stromal cells (MSCs)	miR-146a	TXNIP-dependent action	Diminish VSMC calcification	(103)
	miR-126 and miR-145	Transition of macrophages from a pro-inflammatory and atherogenic phenotype (M1) to an anti-inflammatory and anti-osteogenic phenotype (M2c)	Inhibit thrombosis and calcification	(104)
Fibroblasts	miR155-5p	Reduced vascular angiotensin-converting enzyme, angiotensin II, and proliferating cell nuclear antigen levels	Promote VSMC proliferation and vascular remodeling	(108)

## The Diagnostic and Therapeutic Value of Circulating Exosomes in VC

### Great Diagnostic Potential

Cytologic content of exosomes indicates the biological status of the original cell. As exosomes could modulate their surface signaling molecules to encapsulate selective cargos in different stages of diseases, they may be the most direct and specific non- or semi-invasive targets for early diagnosis of VC. Dynamic expression profiles of exosomal miRNAs can provide clues to the development and progression of cardiovascular disease, including VC. Several studies have reported that miRNA were loaded selectively into EVs (83, 109) since there were distinguished differences in the miRNA profiles of SMCs and their EVs. Moreover, miRNAs were concentrated in exosomes, making up 50% of the total RNA inside. It has been reported that when compared with plasma total miRNAs, EV-derived miRNAs have higher diagnostic efficacy in colon cancer (110). Additionally, exosomes are considered stable and survive in an extreme environment without loss of potency (111). However, direct evaluation of circulation exosomal miRNAs as VC diagnostic or prognostic biomarkers needs to be completed before exosomes be exploited as clinical tools (109).

### Possible Therapeutic Targets

In recent years, researchers have emphasized the great potential of exosomes in therapeutics (82), The exosomes derived from stem cells and MSC have shown cardiovascular protection effects in several disease models, involving the promotion of therapeutic effects in myocardial infarction (112, 113), reduction of myocardial ischemia/reperfusion injury (114, 115), inhibition of pulmonary hypertension (116), and improvement in neurovascular function and plasticity (117). Although these advanced efforts have become one of the key focus in drug development, strong evidence of exosomal therapeutics on VC is still lacking. A number of *in vitro* studies have demonstrated that exosomes exert anti-calcifying effects by decreasing the VSMC phenotype transformation and EndMT, and thus negatively affect hydroxyapatite mineral deposition. And the anti-calcifying effect was mediated by functional cargos such as miR-29b (118), miR-30b/c (118), miR-125b (75), miR-34b/c (119), miR-135a (120), and miR-712 (120).

Those exosomes as well as cargos might be a potential target for gene therapy in VC. However, exosomal miRNAs appear to vary in their ability to promote or inhibit processes depending on the pathological microenvironment. Ulbing et al. (121) found that circulating miR-223 was decreased in patients with CKD. The reduced miR-223 expression has been recognized as a risk factor for VC morbidity. In contrast, when there is elevated inorganic phosphorus, the expression of exosomal miR-223 is upregulated in VSMCs (122). Such a contradiction implies that further studies *in vivo* and clinical use were needed to evaluate the value of exosome-based therapies for VC patients.

It is worth mentioning that although direct evidence is illegible, some studies provide clues that exosomes might offer therapeutic effects on calcifying cardiovascular tissue. Imipramine, an inhibitor of acid sphingomyelinase, can reduce the production of osteoblast-derived calcifying microvesicles (123), and the Ca^2+^ channel blocker verapamil was proved to block calcifying EV biogenesis, matrix vesicle activity, and extracellular matrix (ECM) mineralization, as well as disrupting atheroma formation in the rat aorta (71). Dimerization of the aforementioned sortilin protein also participates in calcification regulation through EV assembling and trafficking, thus inhibiting sortilin homodimer formation is another auspicious EV-associated therapeutic method (124).

Overall, exosomes presenting in the vascular wall microenvironment are not only conceivable candidate biomarkers for VC and its related diseases, but also serve as therapeutic targets.

## Conclusions and Future Perspectives

In this paper, we analyzed the structure of the vascular wall and demonstrate that various cells in the vascular microenvironment are not merely physical structural barriers, but regulators with anti/pro-calcification functions that mediate transcellular regulation by exosomes. Exosomes within the cardiovascular microenvironment also have the capacity of biomarkers and therapeutic targets for VC.

What makes us exciting was that exosomes have been employed as potential carriers for gene therapy (3). Their nano-size and flexibility allow them to cross major biological barriers easily, makes them excellent nanocarriers for delivering chemical drugs and gene drugs, such as siRNAs, miRNAs and antagonist (125). Compared with traditional liposomes, viral vectors, and inorganic nanoparticles, exosomes have relatively low toxicity and immunogenicity. In addition, inherent homing ability of exosomes suggests their potential utility in drug delivery (126, 127). Specifically, exosomes surface could easily be modified, by conjugated with a cell or tissue specific aptamer and cell penetrating peptide which accomplish the goal of precision treatment (128, 129). However, using exosomes as nano-drug delivery implements is currently unable to achieve since its low rate of drug encapsulation (125, 130), how to engineer exosomes so that they are stably expressed once introduced into the recipient body and to avoid off-target effects are important issues to resolve. A big step in the analysis of the formation of exosome compartments would improve understanding of the roles of exosomes and their function in VC. The past decades have witnessed huge advances in molecular imaging, which may provide additional diagnostic methods and technical tools utilizing exosomes to further improve anti-VC therapeutics and diagnostics.

## Author Contributions

All authors listed have made a substantial, direct, and intellectual contribution to the work and approved it for publication.

## Funding

This work was funded by the National Natural Science Foundation of China (Grant Nos. 82070910, 81770881, 82100494, and 82100944), Natural Science Foundation of Hunan Province of China (S2021JJMSXM2818), and Key R&D plan of Hunan Province (2020SK2078).

## Conflict of Interest

The authors declare that the research was conducted in the absence of any commercial or financial relationships that could be construed as a potential conflict of interest.

## Publisher's Note

All claims expressed in this article are solely those of the authors and do not necessarily represent those of their affiliated organizations, or those of the publisher, the editors and the reviewers. Any product that may be evaluated in this article, or claim that may be made by its manufacturer, is not guaranteed or endorsed by the publisher.

## Abbreviations

EV: extracellular vesicle
MVBs: multivesicular bodies
mRNAs: messenger RNAs
miRNAs: microRNAs
ILVs: intraluminal vesicles
MPs: microparticles
ABs: apoptotic bodies
VC: Vascular calcification
CKD: chronic kidney disease
VSMCs: vascular smooth muscle cells
ECs: Endothelial cells
VEGF: vascular endothelial growth factor
PDGF: platelet-derived growth factor
bFGF: basic fibroblast growth factor
IGF-1: insulin-like growth factor 1
NO: nitric oxide
PGI2: prostacyclin
ET: endothelin
EndMT: endothelial–mesenchymal transition
SM22alpha: smooth muscle 22 alpha
OPN: osteopontin
OCN: osteocalcin
BMP-2: bone morphogenetic protein-2
ALP: alkaline phosphatase
RUNX2: Runt-related transcription factor 2
MVs: matrix vesicles
MGP: matrix Gla protein
ROS: reactive oxygen species
TANK: TRAF family member associated NFkappaB activator
CXCL12: Chemokine 12
EMPs: endothelial microparticles
TNF-α: tumor necrosis factor-alpha
HUVECs: human umbilical vein endothelial cells
mTOR: mammalian target of rapamycin
TSG101: Tumor Susceptibility Gene 101
HMGB1: high mobility group box 1
BMDCs: bone marrow dendritic cells
MSCs: Mesenchymal stromal cells
HSCs: hematopoietic stem cells
cMPs: Circulating microparticles
pMPs: platelet-derived microparticles
AC: coronary artery calcification
ACE: angiotensin-converting enzyme
ncRNAs: non-coding RNAs
siRNA: small interfering RNA
ECM: extracellular matrix
Treg: regulatory T
LPS-EK: lipopolysaccharide
BMSC-Exo: bone marrow mesenchymal stem cell-derived exosome.
